# Books Reviewed

**Published:** 1865-12

**Authors:** 


### Books Reviewed.

The Practice of Medicine and Surgery applied to the Diseases and Accidents incident to Women. By William H. Byford, A. M., M. D. Philadelphia: Lindsay & Blakiston, 1865.

It is not long since Prof. Byford favored the profession with a treatise on the chronic inflammation and displacements of the uterus; he has now incorporated this with a comprehensive work upon the medical and surgical diseases of women, and thus furnished one of the most complete and valuable works upon this subject to be found in our own or any other language. The author, with his usual modesty, says that his object has been to furnish students and junior members of the profession a concise, yet sufficiently complete, practical and reliable treatise to meet their wants in every-day practice. Whether he has accomplished this object will be determined by its success alone, and for the verdict thus indicated the author respectfully and hopefully dedicates it to his young friends.

The young as well as the old and tried friends of the author will not fail to appreciate the value of this work. The most recent views on pathology and the approved methods of treatment are embodied in this work; we have not read a work upon this department of medical and surgical practice with so much pleasure and instruction. We believe that whatever is valuable, whatever is really hnown or highly probable will be found comprised within the pages of this work, while the unimportant and undetermined has been silently omitted. One of the excellencies of the work will be found to consist in its highly practical character—the philosophy of diseased action and the modus operandi of cure are propositions which receive undivided attention. The work is appropriately illustrated and published in the best style of Lindsay & Blakiston of Philadelphia. It is for sale in Buffalo by Theodore Butler, 159 Main street, and should certainly be in the possession of every physician.

Lectures on the Diseases of the Stomach, with an introduction on its Anatomy and Physiology. By William Brinton, M. D., F. R. S. From the second English edition. Philadelphia: Lea & Blanchard. 1865.

In this work we have first, the anatomy and physiology of the digestive organs; this chapter, derived from lectures by the author upon physiology, is very handsomely illustrated. Then we have lectures upon the general symptoms of gastric disease; appearances and circumstances connected with examination of the stomach after death; ulcer of the stomach; cancer of the stomach; cirrhotic inflammation or plastic linitis, suppurative linitis, tumors, hypertrophy, atrophy, dilatation, and secondary inflammation; dyspepsia, gastric phthisis; gout in the stomach, etc., etc. These lectures comprise a brief but condensed and quite perfect account of what is at present known concerning diseases of the stomach. The anatomy, physiology, symptoms and treatment are so presented as to make the work a very instructive and popular one with practitioners of medicine. These lectures are upon diseases of the most frequent occurrence, and also of a nature oftentimes to elude detection. It is an every day duty with physicians to decide upon many of these obscure maladies; the work before us cannot fail to prove useful in determining any of the questions which may arise in the management of organic diseases of the stomach as well as the functional derangements of digestion. The work comprises about 300 pages, and is a well written and valuable book, treating upon most important subjects.

